# Identifying immunoreactive proteins in brucellin for enhanced brucellosis diagnosis: a proteomic approach

**DOI:** 10.3389/fmicb.2025.1641710

**Published:** 2025-07-30

**Authors:** Ivanka Krasteva, Mirella Luciani, Federica D’Onofrio, Tiziana Di Febo, Chiara Di Pancrazio, Fabrizia Perletta, Marta Maggetti, Simonetta Ulisse, Luigina Sonsini, Gianluca Orsini, Marco Caporale, Claire Ponsart, Vitomir Djokic, Acacia Ferreira Vicente, Luca Freddi, Nicola D’Alterio, Manuela Tittarelli, Fabrizio De Massis, Flavio Sacchini

**Affiliations:** ^1^Istituto Zooprofilattico Sperimentale dell’Abruzzo e del Molise, Teramo, Italy; ^2^Department of Bioscience and Technology for Food, Agriculture and Environment, University of Teramo, Teramo, Italy; ^3^EU/WOAH & National Reference Laboratory for Brucellosis, Animal Health Laboratory, ANSES/Paris-Est University, Paris, France

**Keywords:** brucellosis, brucellin skin test, proteome, mass spectrometry, serological test

## Abstract

The brucellin skin test (BST) detects brucellosis in animals through a cell-mediated immune response to a protein extract from *B. melitensis* strain 115, which is almost free of lipopolysaccharide. It is highly specific and used to confirm suspected false positive serology results in small ruminants and swine, but not recommended for screening due to low sensitivity. Despite its diagnostic significance, the protein composition of brucellin has not been fully characterized. This study used nLC-ESI-MS/MS analysis and bioinformatics tools to evaluate brucellin’s protein composition and identify immunoreactive proteins. An allergen suspension of purified proteins (free of S-LPS) of EU Standard Brucellin, produced by ANSES, IZS-Teramo (IZSAM) and the former commercialised brucellergene OCB^®^ were used. Proteomic analysis identified 247 (ANSES), 542 (IZSAM) and 183 (OCB) proteins. Two hundred and six proteins (ANSES), 458 proteins (IZSAM) and 156 (OCB) were predicted as potential antigens, and 123 proteins are common to all 3 brucellins examined. Among the 123 proteins common to all three brucellin formulations examined, several key immunodominant proteins previously identified in Brucella research—such as ribosomal L7/L12, outer membrane protein BP26/OMP28, GroEL, and Bacterioferritin—were consistently detected. Their presence across all formulations supports their important role in inducing delayed hypersensitivity and contributing to Brucella pathogenesis. These findings underscore the importance of introducing mass spectrometry analyses as quality control for brucellin batches production and the potential of these proteins as candidates for detecting cellular immunity against *Brucella*. Developing recombinant *Brucella*-allergenic proteins could help in standardizing skin tests, providing reliable allergens favoring disease control and eradication. Moreover, a serological test using these recombinant proteins could improve specificity of current indirect tests for *Brucella* and eliminate false-positive results associated with LPS-based diagnostics.

## 1 Introduction

Brucellosis, a zoonotic disease caused by *Brucella* species, poses significant challenges in veterinary and human health due to its complex pathogenesis and diagnostic limitations (Qureshi et al., 2023). Bacteriological cultures, serological assays (e.g., ELISA), and molecular methods like PCR are used for brucella diagnosis. However, non-specific symptoms and sampling difficulties (such as repeated sampling of the same animal, false positive and negative results, etc.) complicate the process (Aslam et al., 2023). The brucellin skin test (BST) is a diagnostic tool used to detect brucellosis in animals by eliciting a cell-mediated immune response to a protein extract from *B. melitensis* strain 115, with minimal lipopolysaccharide content. It is based on the delayed-type hypersensitivity (DTH) reaction, with results assessed 48 or 72 h after brucellin injection. According to the Commission Delegated Regulation (EU) 2020/689, the BST can only be used in sheep and goats to acquire and maintain the Brucellosis free status (European Commission, 2020). While BST specificity is very high and valuable for suspected false positive small ruminants and swine confirmations, its’ low sensitivity limits the use as a screening method. Specifically, the use of brucellin from the BST is recommended in cases of false positive serological reactions (FPSR) for both *in vivo* (BST) and *in vitro* (IGRA) tests (WOAH, 2022). Additionally, brucellin has also been used in buffaloes and cattle to identify animals vaccinated with RB51 (Tittarelli et al., 2015; De Massis et al., 2005), further supporting its role as diagnostic tool. Although the BST is valuable for brucellosis diagnosis and is considered a highly specific antigen, its exact protein composition remains poorly characterized, limiting its potential for improved sensitivity and specificity. The validation of newly produced antigen batches is based on potency tests carried out in guinea pigs (WOAH, 2022), which rely on measuring the diameter of the cutaneous reaction following intradermal BST injection and are influenced by factors such as experimental conditions, individual immune sensitivity and staff reading. Moreover, there are no true quality controls based on the proteins constituting the mixture, which can result in variability of both the composition and effectiveness of different antigen batches. Recent advances in proteomics have enabled a deeper understanding of the protein components of brucellin, revealing a complex mixture of immunoreactive proteins. Several immunogenic proteins have been identified in brucellin, including: L7/L12 ribosomal protein that plays a role in inducing DTH and is critical for *Brucella* pathogenesis; outer membrane protein BP26/OMP28, known for its antigenic properties and involvement in immune responses; GroEL a heat shock protein that contributes to bacterial stress response and pathogenesis; bacterioferritin involved in iron storage and bacterial survival under stress conditions (Poetsch and Marchesini, 2020). Furthermore, characterization of individual brucellin proteins and identification of key candidates capable of eliciting cellular immunity to *Brucella* are essential. This would support the development of a recombinant protein-based antigen, easier to standardize and potentially applicable for both diagnostics and vaccines applications, thereby contributing to improve disease control and eradication.

Therefore, this study used nanoflow liquid chromatography coupled with electrospray ionization tandem mass spectrometry (nLC-ESI-MS/MS) and bioinformatics tools to comprehensively evaluate the protein composition of various brucellin formulations. Thus, the objective of this study was to characterize and compare different brucellin preparations, to establish the minimal protein profile associated with biological efficacy to be used as quality control for new antigen batches. The potential of the identified proteins in next-generation diagnostics or as candidate antigens for vaccines is also discussed.

## 2 Materials and methods

### 2.1 Bacterial strain and growth conditions

An allergen suspension of purified proteins (free of S-LPS) of EU Standard Brucellin (batch 2011-01), produced by ANSES, an experimental batch of brucellin produced by Istituto Zooprofilattico Sperimentale dell’Abruzzo e del Molise (IZSAM) and the former commercialised brucellerge OCB^®^ (Zoetis, France) were used.

The ANSES EU brucellin was produced for standardisation of future batches, in concentration of 2,000 units per ml. It is a pool of 12 different commercial brucellin batches, which passed tests of purity, appearance, sensitising effect and potency in guinea pig model. Activity was compared to reference Brucellin INRA at same concentration, by ANSES team, according to WOAH Manual for the standardisation of diagnostic tests and vaccines (diagnostic tests and vaccines for terrestrial animals) (WOAH, 2022). The experimental batch of brucellin IZSAM was produced according to De Massis et al. (2005) using the *B. melitensis* B115 strain (De Massis et al., 2005). Briefly, the lyophilized strain of *Brucella melitensis* B115 was reconstituted with ultrapure sterile water and plated on glycerol dextrose agar. After 48 h of aerobic incubation at 37 ± 2°C, single colonies were collected and inoculated into 5 test tubes containing glycerol dextrose agar slopes. Following an additional 48-h incubation, the bacterial pellet from each tube was suspended in sterile saline solution, checked for purity using Gram staining, and used to inoculate 5 Roux flasks containing glycerol dextrose agar. After 96 h of incubation at 37 ± 2°C, the bacterial pellet was collected by centrifugation in sterile saline solution, assessed for purity and phase, and subsequently inactivated in a water bath at 70 ± 2°C for 90 min. The suspension was centrifuged at 10,000 g for 30 min at +4 ± 2°C, and the supernatant was discarded. The pellet was resuspended in ultrapure sterile water (1:40 w/v), agitated for 1 h, and the pH was adjusted to 9.6 using 0.5 N NaOH, followed by autoclaving at 100°C for 120 min. After cooling to room temperature, the suspension was centrifuged again, and the supernatant was supplemented with 40% trichloroacetic acid (TCA) (1:10 v/v) and incubated at room temperature for 24 h. The resulting precipitate was resuspended in 1% TCA (1:10 v/v) and incubated for another 24 h, then washed twice with 5% NaCl containing 0.5% phenol (85%) until a final pH of 2.6 ± 0.1 was reached. The pellet was centrifuged again, weighed, and resuspended in phosphate buffer (pH 11) to achieve a final pH of 7.2 (approximately 3 mL of buffer per gram of pellet). The suspension was agitated for 1 h, diluted 1:20 in phosphate buffer (pH 7.2), and sterile-filtered using 0.22 μm pore filters.

### 2.2 Tryptic digestion

All three brucellin protein suspensions were processed using the Filter-Aided Sample Preparation (FASP) protocol. Thirty μg of protein extracts were subjected to reduction with DTT, then alkylated using 50 mM iodoacetamide. Protein digestion was carried out with trypsin, added at a substrate-to-enzyme ratio of 50:1 (w/w), and incubated at 37°C overnight. The enzymatic activity was terminated by acidification with 10% formic acid. The resulting peptide mixtures were then desalted using a C18 column before being subjected to mass spectrometry analysis.

### 2.3 Mass spectrometry analysis (nLC-ESI-MS/MS)

Four μL of extracted peptides from each sample were analysed in triplicate by Liquid Chromatography Tandem Mass Spectrometry (LC-MS/MS) using an Easy-nLC 1200 nano System (Thermo Fisher Scientific) coupled to an Orbitrap Q-Exactive mass spectrometer (Thermo Fisher Scientific). Peptides were loaded on a PepMap pre-column (75 μm i.d., 200 mm L, Thermo Fisher Scientific) and separated on an EASY Spray C18 analytical column (50 μm i.d., 150 mm L., 2 μm ps, Thermo Fisher Scientific). A chromatographic gradient was employed over 97 min. The mass spectrometer operated at 70,000 resolution in full scan mode and used data dependent acquisition (DDA) for shotgun proteomics. The top 12 method was applied, meaning that 12 most abundant peptide ions from first readout were selected for fragmentation and identification in tandem mass spectrometry (MS/MS). The choice of 12 most abundant peptides optimised the depth and the speed of analysis, ensured the most efficient fragmentation of these ions as well as reduced redundancy in high-throughput analysis. Raw data were processed with Proteome Discoverer (version 2.5, Thermo Fisher Scientific) searching against *Brucella melitensis* B115, assuming a fragment ion mass tolerance of 0.02 Da and a parent ion tolerance of 10 ppm; specified enzyme was trypsin; carbamidomethylation of cysteine was set as a fixed modification; oxidation of methionine and acetylation of the N-terminus of proteins were set as variable modifications. Only proteins detected in at least 2 out of 3 biological replicates of the same brucellin formulation were considered for analysis.

### 2.4 Bioinformatics analysis

Data generated by mass spectrometry were then submitted for bioinformatics analysis for protein identification and selection. A combination of softwares was applied to identify cytosolic and non-cytosolic proteins. LipoP 1.0 Server (Juncker et al., 2003) was used for prediction of lipoprotein signal peptides; TMHMM Server version 2.0 was predictive of transmembrane helices (567–580) (Krogh et al., 2001) and SignalP 4.1 Server (Petersen et al., 2011) was applied for signal peptides prediction. PSORTb version 3.0.2 (Yu et al., 2010) and CELLO version 2 (Yu et al., 2004; Yu et al., 2006) predicted subcellular localization. Virulent Pred and VaxiJen v.2.0 tools were used to identify potential immunogenic candidates. Virulent Pred is a bacterial virulent protein prediction method that uses bi-layer cascade support vector machine (Garg and Gupta, 2008). Vaxijen is an alignment-independent server for antigens and proteins physicochemical properties prediction (Doytchinova and Flower, 2007) and the proteins with adhesion score higher than 0.4 were considered as antigens. The potential brucellin immunogenic proteins, identified through bioinformatics analyses, were screened using BLASTp to assess sequence similarity with other *Brucella* species and cross-reactive bacteria. Within the *Brucella* genus, *B. melitensis*, *B. ovis*, *B. abortus*, and *B. suis* were considered. Cross-reactive bacteria included *Pseudomonas aeruginosa*, *Bordetella bronchiseptica*, *Actinobacillus equuli*, *Streptococcus* spp., *Staphylococcus* spp., *Moraxella* spp., *Salmonella enterica* spp., *Yersinia enterocolitica* O:9, *Campylobacter* spp., *E. coli* and *Francisella* spp. (Camacho et al., 2009) as well as the environmental bacterium *Ochrobactrum anthropic* and *Ochrobactrum intermedium* and plant-associated bacteria such as *Rhizobium leguminosarum*, *Rhizobium/Agrobacterium group*, and *Rhizobium tropici* (WOAH, 2022). Non-homologous proteins were identified based on an identity and/or coverage threshold of less than 95% for *Brucella* species and less than 35% for cross-reactive bacteria.

### 2.5 Functional annotation and pathway enrichment analysis

The potential immunogenic proteins commonly identified across Brucellin ANSES, IZSAM, and Brucellergene OCB^®^ were subjected to functional annotation using the KEGG Automatic Annotation Server (BlastKOALA). Protein sequences were uploaded in FASTA format, and the annotation was performed against the KEGG GENES database (prokaryotes + eukaryotes) with taxonomic specification for *Brucella melitensis*. The resulting KEGG Orthology (KO) identifiers were used to assign functional categories and metabolic pathways.

## 3 Results

**Figure 1:**
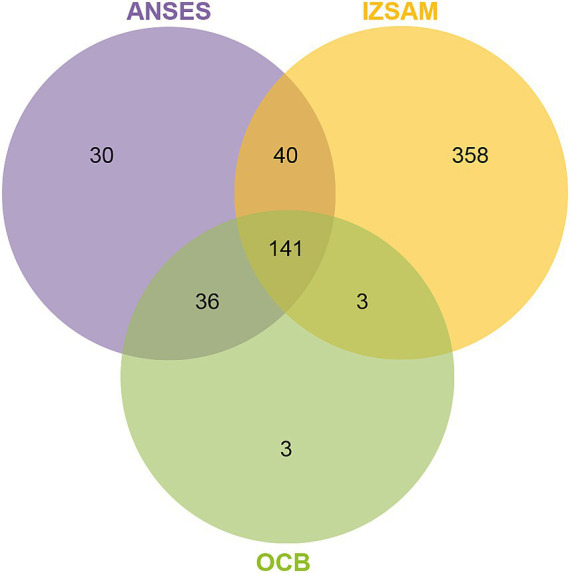
Venn diagram illustrating the overlap of proteins identified by LC-MS/MS in three brucellins formulations: ANSES, IZSAM, and the commercial brucellergene OCB^®^. A total of 141 proteins were shared among all three formulations. In total, 247 proteins were identified in ANSES, 542 in IZSAM, and 183 in OCB.

**Figure 2:**
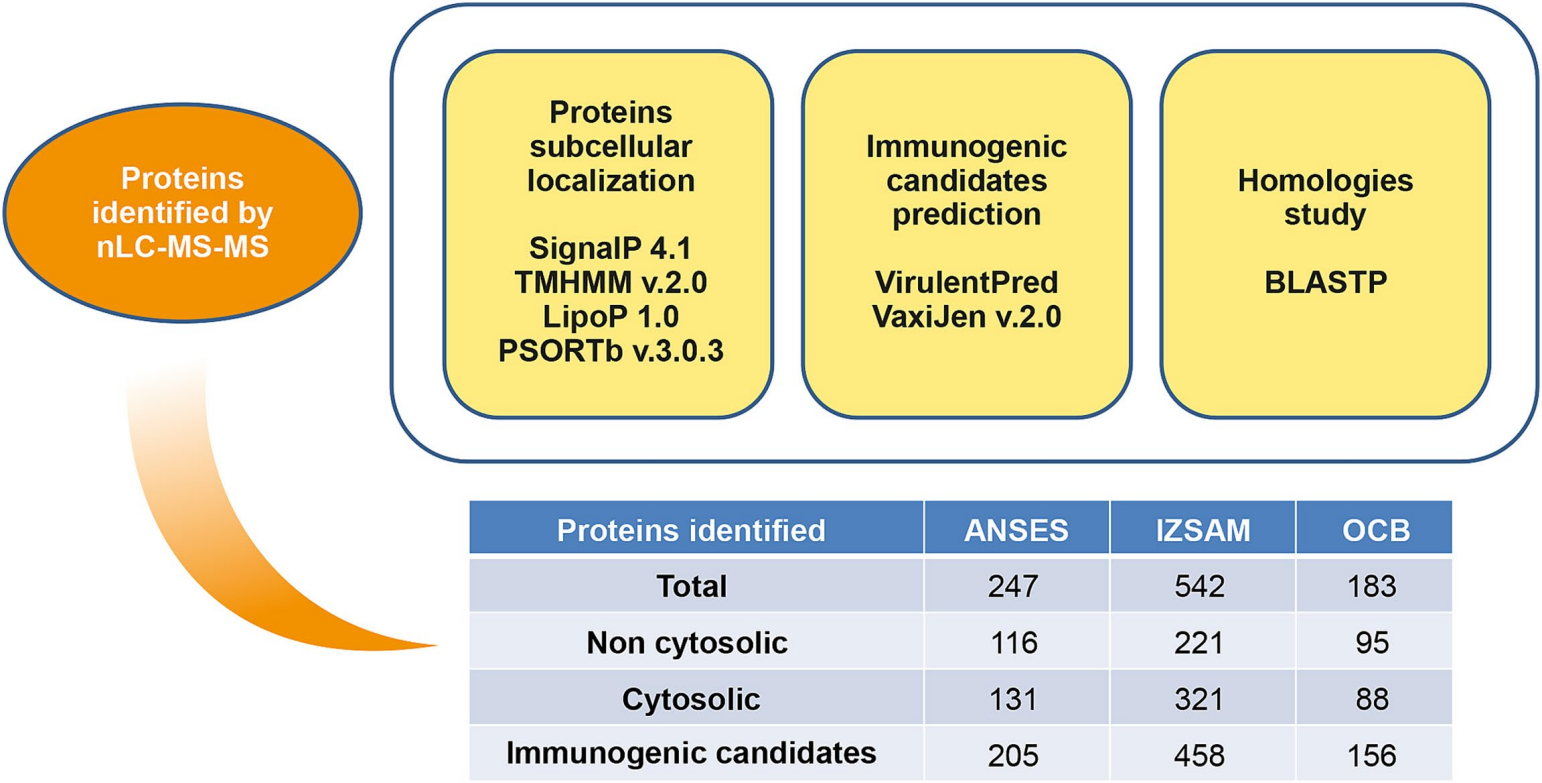
Overview of bioinformatics tools used for prediction of protein candidates.

**Figure 3:**
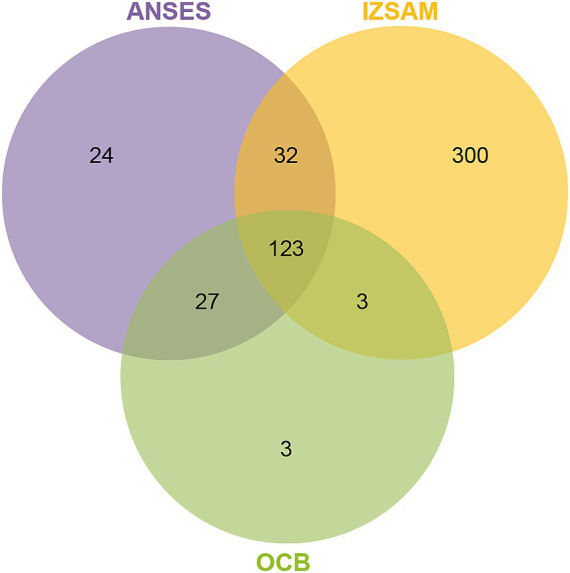
Venn diagram of three brucellin formulations (ANSES, IZSAM, and brucellergene OCB^®^), showing the overlap of immunogenic proteins identified by LC-MS/MS and predicted by VirulentPred and VaxiJen v2.0 (threshold >0.4). A total of 205 proteins in ANSES, 458 in IZSAM and 156 in OCB^®^ were classified as potential antigens. Among these, 123 immunogenic proteins were common to all three formulations. Selected examples of these shared proteins, such as L7/L12, outer membrane protein BP26/OMP28, GroEL, and Bacterioferritin are indicated in the diagram. A complete list is provided in Supplementary Tables 1–3.

The protein suspensions of brucellin produced by ANSES, IZSAM and the brucellergene OCB^®^ were analysed by mass spectrometry analysis and 247, 542 and 183 proteins were identified, respectively with 141 common proteins (Figure 1). One year later, brucellin IZSAM was reanalyzed to assess its stability, revealing the presence of 520 proteins. Prediction of subcellular localisation by LipoP Server, TMHMM Server version 2, SignalP 4.1 Server and PSORTb version 3.0.3 (Supplementary Table 1) classified 116 (47%) (ANSES) 221 (41%) (IZSAM) and 95 (52%) (OCB) as non-cytoplasmic. Moreover, the identified proteins were analysed by Virulent Pred and VaxiJen v.2.0 tools in order to identify potential immunogenic candidates (Supplementary Table 2). The workflow used to predict protein candidates is illustrated in Figure 2. Two hundred and five proteins (ANSES), 458 proteins (IZSAM) and 156 proteins (OCB) were identified as potential antigens with threshold greater than 0.4 (Figure 3). One hundred and twenty-three of those proteins are common to all 3 examined brucellin formulations, with 113 of them also present in the reanalyzed brucellin IZSAM (Supplementary Table 2). Only proteins detected in at least two out of three biological replicates of the same brucellin formulation were considered for analysis. Among the 123 proteins commonly identified across all formulations, 101 were detected in all 3 replicates for IZSAM, 107 for OCB and 122 for ANSES.

**Figure 4:**
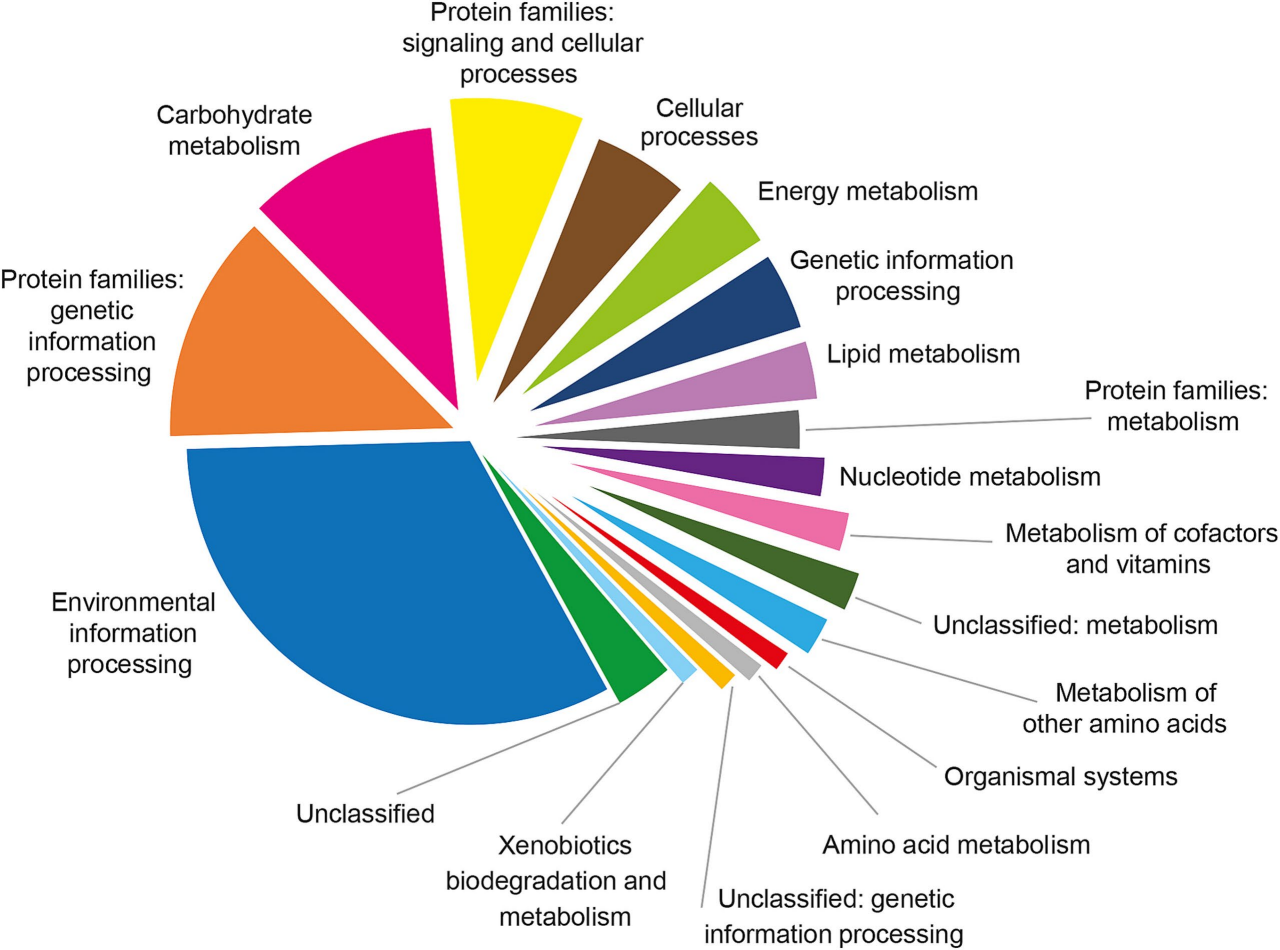
Functional classification of the 123 proteins shared among Brucellin ANSES, Brucellin IZSM, and Brucellergene OCB^®^ based on KEGG pathway annotation using BlastKOALA. The pie chart illustrates the distribution of proteins across functional categories and pathways, with the majority associated with environmental information processing, carbohydrate metabolism, and protein families involved in genetic and cellular processes.

Pathway enrichment analysis revealed that the Brucellin proteome was predominantly associated with key metabolic routes, including carbohydrate metabolism, energy metabolism, lipid metabolism, nucleotide metabolism, metabolism of other amino acids, and the metabolism of cofactors and vitamins as showed in Figure 4.

BLAST was then used to assess the similarity between the 123 common potential antigenic brucellin proteins, also common with other *Brucella* species, as well as cross-reactive bacteria (Supplementary Table 3). All brucellin proteins resulted homologous to *B. abortus*, *B. suis*, and *B. ovis*. Eight proteins were non-homologous to cross-reactive bacteria *Pseudomonas aeruginosa*, *Bordetella bronchiseptica*, *Actinobacillus equuli*, *Streptococcus* spp., *Staphyloccus* spp., *Moraxella type*, *Salmonella* spp., *Campylobacter* spp. and *Francisella* spp. (Table 1). Among them is the protein peptidoglycan hydrolase inhibitor PhiA (WP_004684650.1), which in *Brucella abortus* inhibits SagA, a lytic transglycosidase, balancing peptidoglycans remodeling. Its deletion disrupts lipopolysaccharide synthesis but increases replication, highlighting a link between peptidoglycan remodeling and growth rate (Del Giudice et al., 2019). The remaining 7 proteins are uncharacterised: two proteins contain a domain of unknown function DUF1192 (WP_002964825.1) and DUF1176 (WP_002964611.1); and 5 proteins are hypothetical (ENT65610.1, ENT65708.1, ENT68135.1, WP_002964988.1, WP_002965314.1). Compared to *Yersinia enterocolitica* O:9, 59 proteins were identified as non-homologous. Among them are well known proteins that play a critical role in *Brucella* virulence, such as outer membrane protein BP26/OMP28, EipB, and one of the ABC transporters’ substrate-binding proteins. Twenty-eight hypothetical proteins were also found, but their role in virulence is yet unknown.

**Table 1:** List of non-homologs proteins to cross-reactive bacteria, (*Pseudomonas aeruginosa*, *Bordetella bronchiseptica*, *Actinobacillus equuli*, *Streptococcus* spp., *Staphylococcus* spp., *Moraxella type*, *Salmonella* spp., *Campylobacter* spp. and *Yersinia enterocolitica* O:9).

Entry	Protein names	Function
ENT65610.1	Hypothetical protein D627_02230	Unknown
ENT65708.1	Hypothetical protein D627_02331	Unknown
ENT68135.1	Hypothetical protein D627_01855	Unknown
ENT68740.1	Hypothetical protein D627_00252	Unknown
WP_002964611.1	DUF1176 domain-containing protein	Unknown
WP_002964825.1	DUF1192 domain-containing protein	Unknown
WP_002964988.1	Hypothetical protein	Unknown
WP_002965314.1	Hypothetical protein	Unknown
WP_004684650.1	Peptidoglycan hydrolase inhibitor PhiA	Peptidoglycan remodeling

## 4 Discussion

In Europe, animal brucellosis is still present in the south of the continent currently, the most specific confirmatory indirect diagnostic tool for identifying Brucella-positive animals is BST. This test is based solely on cellular immunity and is recommended for use only in small ruminants. However, based on the number of countries in which this method is applied and annual tests used for pharmaceutical companies the production of brucellin is becoming more and more unjustified. One of the main reasons is the costly standardisation of brucellin formulation and activity testing in guinea pigs. Therefore, we used proteomic shotgun approach to analyse three available brucellin formulations to identify key components potentially responsible for its immunogenic activity, which could be used for future development of *Brucella* indirect diagnostic tools (Poetsch and Marchesini, 2020).

The analysis of brucellin protein suspensions from ANSES, IZSAM, and the brucellergene OCB^®^ using mass spectrometry identified a substantial number of common proteins across the different samples. Mass spectrometry analysis revealed 247 different proteins in the ANSES sample, 542 in the IZSAM sample, and 183 in the brucellergene OCB^®^. Among these, a significant proportion were classified as non-cytoplasmic based on subcellular localisation predictions. A considerable number of proteins were predicted as potential antigens using tools like VirulentPred and VaxiJen v.2.0. Specifically, 205 proteins from ANSES, 458 from IZSAM, and 156 from OCB met this criterion. There were 123 common potential antigenic proteins between all three brucellin formulations analysed. Our findings highlight the heterogeneity of protein composition among brucellins from different producers. Notably, the ANSES brucellin analyzed was a pooled sample derived from 12 batches produced according to the WOAH Manual, while OCB^®^ and IZSAM were represented by single batch preparations. This variability underscores the importance of the introduction of quality controls aimed to assess the protein composition of the different brucellin antigens/batches and demonstrates that proteomic analysis can be used as a quality control tool to ensure the standardisation and reproducibility of the antigens. For brucellin ANSES and brucellergene OCB^®^, the potency of the antigens was validated using the guinea pig model (WOAH, 2022). This suggests that the protein (s) responsible for DTH falls within the 123 common proteins, that were detected in all the 3 antigens.

Moreover, the enrichment of pathways related to carbohydrate, energy, lipid, nucleotide metabolism, metabolism of other amino acids, and cofactors/vitamins, suggests the presence of immunogenic proteins involved in essential bacterial functions. Several enzymes within these pathways, such as glycolytic and lipid-associated proteins, are known to act as antigens or pathogen-associated molecular patterns (PAMPs), capable of stimulating innate immune receptors and promoting antigen presentation via MHC molecules. These proteins may enhance T-cell activation, particularly favouring Th1-type responses characteristic of delayed-type hypersensitivity (DTH). Additionally, metabolic processes such as arginine and cofactor biosynthesis can modulate macrophage function and cytokine production, further contributing to a robust cell-mediated immune response. Collectively, these findings support the immunostimulatory potential of Brucellin components in eliciting protective cellular immunity against *Brucella* spp.

BLAST analysis showed that 123 common antigenic proteins are homologous to those found in other *Brucella* species (*B. abortus*, *B. suis*, *B. ovis*) as well as environmental bacteria like *Ochrobactrum anthropi* and plant pathogens/symbionts such as *Rhizobium leguminosarum*. This is it be expected, since genetic similarities between *Brucella* spp., especially core species such as *B. melitensis* and *B. abortus* used for brucellin production, and *Ochrobactrum* spp. are very high [average nucleotide identity (ANI) higher than 80%] (Ashford et al., 2020). While the possibility of diagnostic interference theoretically exists due to genetic similarities between *Brucella* and other bacteria, there are currently no studies documenting specific diagnostic interference effects between *Ochrobactrum*, Rhizobium, and brucellosis diagnosis in cattle. Nine proteins did not show homology with cross-reactive bacteria including *Pseudomonas aeruginosa*, *Bordetella bronchiseptica*, etc., suggesting they might be specific to brucellosis diagnostics or vaccine development. For *Yersinia enterocolitica* O:9, 59 proteins were identified as non-homologous. Among them are well known proteins that play a critical role in *Brucella* virulence such as the outer membrane protein BP26/OMP28, EipB and one of the ABC transporter substrate-binding proteins. Additionally, 28 hypothetical proteins were found and their potential role in virulence warrants further investigation.

In this study, several well-known proteins recognized for their immunogenicity and ability to induce DTH were identified in all 3 brucellins exanimated. These proteins were also present in the reanalyzed brucellin IZSAM. According to bioinformatics analysis all these proteins are immunogenic. A more focused inspection of key proteins such as L7/L12, BP26/OMP28, GroEL, and Bacterioferritin reveals their critical roles in brucellosis diagnosis and vaccine development. The L7/L12 protein is particularly noteworthy for its ability to induce strong DTH, a property dependent on post-translational modification, as noted (Bachrach et al., 1997) which is crucial for brucellosis diagnostics. The homologous of this protein is also a major component of tuberculin purified protein derivative (PPD) and is recognized as a heat-resistant protein capable of eliciting robust DTH reactions (Akbari et al., 2019; Huy et al., 2021; Kitaura et al., 1999; Zhu et al., 2020). The use of L7/L12 in diagnostic formulations, such as skin tests, could improve sensitivity, reduce false negative results and enable more accurate field detection of brucellosis in livestock. BP26/OMP28 is a highly immunogenic antigen in *Brucella* spp., widely recognized for its potential in both diagnostic applications and vaccine development. Moreover, vaccination of mice with recombinant OMP28 (rOMP28) greatly increased IFN-γ, IL-2, and TNF-α levels (Im et al., 2018; Kaushik et al., 2010). In addition, structural and regulatory proteins such as 50S ribosomal protein L9 (Naganathan et al., 2015), ribosome-recycling factor (Hovingh et al., 2016) and transcription elongation factor GreA (Yin et al., 2023), may act as immunogens, due to their intracellular concentrations and essential roles in bacterial survival under host-imposed stresses. Several other proteins, critical to the cellular functions of *Brucellae* and its interaction with the host immune system, were identified. These include chaperonins, GroEL, superoxide dismutase, DNA-binding proteins and various ABC transporter substrate-binding proteins. Among the ABC transporter substrate-binding proteins, the siderophore ABC transporter plays a critical role in *Brucella* virulence by facilitating iron acquisition (Eskra et al., 2012) and is a potential vaccine candidate (Malik et al., 2023). Experimental studies have shown that purified GroEL can induce significant DTH reactions and stimulate T-cell responses, particularly Th1-type immunity, which is critical for effective defense against intracellular pathogens like *Brucella* (Pais et al., 1998). In the study Oliveira et al. (1996) indicates that recombinant *Brucella abortus* GroEL protein can prime CD4^+^ T cells to proliferate and secrete interferon-gamma (IFN-γ), a hallmark of Th1-type immune responses associated with cellular innate immunity and DTH. In *Brucella*, GroEL works with its co-chaperone GroES to form a nano-cage that encapsulates misfolded proteins, facilitating their proper folding through ATP-dependent cycles. However, our analysis did not identify GroES as one of prominent component of either of three analysed brucellin formulations. Bacterioferritin, a conserved protein in *Brucella*, plays a vital role in iron homeostasis and protection against oxidative stress. It is an important antigen expressed during infection and is recognized by the host immune system. Previous research has highlighted its role as a major T-cell antigen in *B. melitensis*, with studies such as those by Oliveira et al. (1996) identifying bacterioferritin as a significant component of the B115 protein preparation (Denoel et al., 1997). This underscores its role in eliciting cellular immune responses and its potential as a indirect diagnostic target. Furthermore, Al-Mariri et al. (2001) demonstrated that vaccination with recombinant bacterioferritin, combined with pG oligodeoxynucleotides as an adjuvant, induced robust immune responses and provided protection against *Brucella abortus* 544 in mice. This supports its inclusion in diagnostic formulations like brucellin to enhance DTH detection in infected animals. The inclusion of bacterioferritin in the B115 protein preparation highlights its critical role as an immunodominant protein, advancing the understanding of *Brucella* pathogenesis and offering new avenues for diagnostics and vaccine development. As a T-cell antigen, bacterioferritin holds promise not only as a diagnostic tool but also as a potential therapeutic target. EipB, another conserved and immunogenic protein in *Brucella* species, is a periplasmic protein. It plays a crucial role in maintaining cell envelope integrity that not only contributes to the virulence of *Brucellae* but also influences the host’s immune response by inducing antibody production and potentially modulating T helper cell activity. This indicates that EipB is significant not only for its role in pathogenesis but also for its impact on the immune dynamics during infection (Herrou et al., 2019). Other proteins identified in all three brucellin formulations, such as Dps, cold-shock proteins, IalB family, and PPIases play crucial roles in *Brucella* virulence and intracellular survival. Dps manipulates host iron homeostasis and protects against oxidative stress (Hop et al., 2023). Cold-shock proteins enable adaptation to environmental stress, enhancing persistence (Wang et al., 2014). IalB (invasion-associated-locus B) family proteins contribute to host cell invasion and immune system engagement in intracellular pathogens such *Bartonellae* (Jin et al., 2023), and they help adaptation to host cellular environment of *Brucellae* (Comerci et al., 2023). Peptidylprolyl isomerase assist in proper folding of immunogenic proteins, indirectly enhancing immune detection (Pandey et al., 2017). Forty-four hypothetical proteins and domain-specific proteins were also identified as components of all three brucellin formulations. These include DUF1176, DUF1192 and SPOR domain-containing proteins, still underexplored in *Brucellae* but based on homology probably are involved in bacterial cell wall maintenance, remodeling, division and other processes, they could also hold significant potential for virulence and DTH induction. These proteins may broaden the scope of recombinant protein technologies for brucellin improvement. Interestingly, only in brucellergene OCB^®^, zinc ABC transporter substrate-binding protein ZnuA was identified, which is known for its critical role in bacterial virulence in mice, making it a promising target for vaccine development (Kim et al., 2004). Several immunologically significant proteins uniquely identified in brucellin produced by IZSAM have shown promise as vaccine candidates or as key factors in understanding *Brucella* pathogenesis. Outer membrane proteins (OMPs) are pivotal in *Brucella* virulence and immune modulation, especially activation of TLRs 2 and 4. Among these, OmpA family, particularly recombinant OmpA, have shown promise in enhancing humoral and cell-mediated IgG1 immune responses, IgG2a, TNF-α, and IL-12, suggesting their potential as subunit vaccine candidates (Simborio et al., 2016). Similarly, Omp10, Omp19 and Omp31 have been identified as essential for virulence (Tibor et al., 2002), and their attenuation observed in strains lacking these OMPs highlights their potential as a protective antigens (Verdiguel-Fernández et al., 2020). Proteins that directly modulate the immune system, also found in brucellin, further emphasize the adaptability of *Brucella*. Pal has been shown to induce TNF-α and IFN-γ production in macrophages, and its absence results in reduced bacterial proliferation, stress resistance, and virulence, underlining its importance in pathogenicity (Chen et al., 2022). Similarly, elongation factor G has been identified as a potent immunoreactive protein capable of eliciting robust immune responses, making it a promising vaccine candidate (Zhao et al., 2011). LemA family proteins also show immunological significance, being identified as serodominant (Nandini et al., 2023). Proteins involved in stress tolerance and intracellular survival further underscore *Brucella*’*s* ability to adapt to hostile environments. Group 2 porins, for example, are effective in inducing delayed-type hypersensitivity through robust cell-mediated immune responses (Mahajan et al., 2005). ClpP, which is essential for bacterial growth, has been implicated in stress tolerance and virulence, highlighting its significance in the pathogen’s survival strategy (Sun et al., 2023). These proteins, taken together not only enhance our understanding of *Brucella* pathogenesis. Their ability to modulate immune responses, promote stress survival, and induce host pathogen interaction (protective immunity) underscores their significance. However, how to standardize their percentage in brucellin while retain or improve formulation efficacy still remains to be determined. At the same time, future research focused on these targets could lead to improved diagnostics and vaccines against *Brucella* infections.

Another path would be to use recombinant form of identified immunogenic protein(s), which induce DTH, for the production of brucellin. The use of recombinant proteins derived from *Brucellae* in diagnostic formulations would offer significant advantages over traditional methods increasing antigen standardization with positive impact on sensitivity and specificity of brucellin based tests. Recent studies have shown that recombinant *Brucella* proteins, such as L7/L12, Omp16, Omp19, and Omp28, elicit strong immune responses when used combined in subunit vaccines (Huy et al., 2021). Recombinant proteins also mitigate the risks associated with manipulation of live *Brucella* strains required for antigen preparations, ensuring higher safety for personnel and mitigating risks of laboratory accidents. Furthermore, recombinant versions of these proteins could provide a standardized and scalable solution, improving the consistency and efficacy of diagnostic skin tests while reducing reliance on whole-cell antigen preparations. L7/L12, BP26/OMP28, GroEL, and bacterioferritin are crucial players in *Brucella*’s ability to evade the host immune system while simultaneously serving as valuable diagnostic and vaccine candidates.

This study made available the composition of three currently available brucellin formulations and demonstrated the efficacy of proteomics as new tools for quality control of new bacteriological diagnostic approaches. These results should be combined with *in vivo* testing on selected target species for initial validation and comparison of new brucellin formulations. Future studies should explore the immune responses elicited by these proteins and their combinations to validate their roles in inducing DTH and their suitability for integration into brucellin as recombinant molecules.

## 5 Conclusion

The proteomic analysis of brucellin protein suspensions from ANSES, IZSAM, and brucellergene OCB^®^ has offered valuable insights into the composition of these formulations and participation of immunogenic *Brucella* peptides, revealing a diverse array of proteins that could be useful for improved brucellosis diagnostics and vaccine development. The identification of significant immunogenic proteins along with various outer membrane and regulatory proteins, underscores their essential roles in DTH responses and their potential as candidates for recombinant brucellin formulations. The finding of 123 common antigenic proteins across the three brucellin formulations, in addition to proteins unique to each preparation, emphasizes shared immunodominant targets but also an important variability of protein composition that calls for standardization of antigen formulations and application of appropriate quality controls. Crucially, the identification of proteins that do not share homology with cross-reactive bacteria presents an opportunity to improve the specificity of current indirect test for *Brucella*, mitigating false positives associated with LPS-based diagnostics. Taken together our findings pose the scientific basis for improvement of current tools for brucellosis control and eradication. Even though Brucellin is produced following standardized inactivation protocols, the protein content can still vary between batches—something that might influence how well the antigen performs in diagnostic tests. To explore this, we applied a proteomic shotgun approach to better understand its composition and to identify key immunoreactive proteins—specifically, the ribosomal protein L7/L12 and the cytoplasmic protein P39—which are known to trigger a delayed-type hypersensitivity response in infected animals. These proteins could act as useful markers for future quality control checks. We’re fully aware that in regions where brucellosis is still endemic, resources are limited. That’s why our goal is not to replace existing WOAH guidelines, but rather to suggest some complementary tools. For instance, an initial proteomic analysis could help establish a reference composition, while simple and cost-effective immunoassays focused on those key antigens could offer a practical way to validate batches over time (Wareth et al., 2020; Naseer et al., 2023).

## Data Availability

The datasets presented in this study can be found in online repositories. The names of the repository/repositories and accession number(s) can be found at: https://www.ebi.ac.uk/pride/archive/, PXD063758.

## References

[ref1] Akbari R. Sekhavati M. H. Bahrami A. Majidzadeh Heravi R. Yousefi S. (2019). Production of Brucella lumazine synthase recombinant protein to design a subunit vaccine against undulant fever. Arch. Razi Inst. 74, 1–6. doi: 10.22092/ari.2019.117997, PMID: 31013002

[ref2] Al-Mariri A. Tibor A. Mertens P. De Bolle X. Michel P. Godefroid J.. (2001). Protection of BALB/c mice against *Brucella abortus* 544 challenge by vaccination with bacterioferritin or P 39 recombinant proteins with CpG oligodeoxynucleotides as adjuvant. Infect. Immun. 69, 4816–4822. doi: 10.1128/IAI.69.8.4816-4822.2001, PMID: 11447155 PMC98569

[ref3] Ashford R. T. Muchowski J. Koylass M. Scholz H. C. Whatmore A. M. (2020). Application of whole genome sequencing and pan-family multi-locus sequence analysis to characterize relationships within the family Brucellaceae. Front. Microbiol. 11:1329. doi: 10.3389/fmicb.2020.01329, PMID: 32760355 PMC7372191

[ref4] Aslam M. A. Mehnaz S. Fatima T. Ather A. S. Tehreem A. Haq S. U.. (2023). Brucellosis: a global challenge. Faisalabad, Pakistan: Zoonosis, Unique Scientific Publishers, 432–442.

[ref5] Bachrach G. Banai M. Fishman Y. Bercovier H. (1997). Delayed-type hypersensitivity activity of the Brucella L7/L12 ribosomal protein depends on posttranslational modification. Infect. Immun. 65, 267–271. doi: 10.1128/iai.65.1.267-271.1997, PMID: 8975922 PMC174586

[ref6] Camacho C. Coulouris G. Avagyan V. Ma N. Papadopoulos J. Bealer K.. (2009). BLAST+: architecture and applications. BMC Bioinformatics 10:421. doi: 10.1186/1471-2105-10-421, PMID: 20003500 PMC2803857

[ref7] Chen Y. Fu Y. Kong L. Wang F. Peng X. Zhang Z.. (2022). Pal affects the proliferation in macrophages and virulence of Brucella, and as mucosal adjuvants, provides an effective protection to mice against *Salmonella enteritidis*. Curr. Microbiol. 80:2. doi: 10.1007/s00284-022-03107-w, PMID: 36418790 PMC9684781

[ref8] Comerci D. J. Marchesini M. Rios N. (2023). Characterization of IalB family protein in Brucella abortus. In the proceedings of Brucellosis 2022 International Research Conference; 16–19 Sep 2022; Giulianova (Teramo). Italy. Teramo, Italy: Istituto Zooprofilattico Sperimentale dell’Abruzzo e del Molise “G. Caporale”. Available online at: https://www.veterinariaitaliana.izs.it/index.php/BRUC/article/view/2789

[ref9] De Massis F. Giovannini A. Di Emidio B. Ronchi G. F. Tittarelli M. Di Giannatale E.. (2005). Use of the complement fixation and brucellin skin tests to identify cattle vaccinated with *Brucella abortus* strain RB51. Vet. Ital. 41, 291–299, PMID: 20437382

[ref10] Del Giudice M. G. Romani A. M. Ugalde J. E. Czibener C. (2019). PhiA, a peptidoglycan hydrolase inhibitor of Brucella involved in the virulence process. Infect. Immun. 87:e00352-19. doi: 10.1128/IAI.00352-19, PMID: 31182616 PMC6652757

[ref11] Denoel P. A. Vo T. K. Weynants V. E. Tibor A. Gilson D. Zygmunt M. S.. (1997). Identification of the major T-cell antigens present in the *Brucella melitensis* B115 protein preparation, Brucellergene OCB. J. Med. Microbiol. 46, 801–806. doi: 10.1099/00222615-46-9-801 9291893

[ref12] Doytchinova I. A. Flower D. R. (2007). VaxiJen: a server for prediction of protective antigens, tumour antigens and subunit vaccines. BMC Bioinformatics 8:4. doi: 10.1186/1471-2105-8-4, PMID: 17207271 PMC1780059

[ref13] Eskra L. Covert J. Glasner J. Splitter G. (2012). Differential expression of iron acquisition genes by *Brucella melitensis* and *Brucella canis* during macrophage infection. PLoS One 7:e31747. doi: 10.1371/journal.pone.0031747, PMID: 22403618 PMC3293887

[ref14] European Commission (2020). Commission Delegated Regulation (EU) 2020/689 of 17 December 2019 supplementing Regulation (EU) 2016/429 of the European Parliament and of the Council as regards rules for surveillance, eradication programmes, and disease-free status for certain listed and emerging diseases. 211–379. Available online at: http://data.europa.eu/eli/reg_del/2020/689/oj

[ref15] Garg A. Gupta D. (2008). Virulent Pred: a SVM based prediction method for virulent proteins in bacterial pathogens. BMC Bioinformatics 9:62. doi: 10.1186/1471-2105-9-62, PMID: 18226234 PMC2254373

[ref16] Herrou J. Willett J. W. Fiebig A. Czyż D. M. Cheng J. X. Ultee E.. (2019). Brucella periplasmic protein Eip B is a molecular determinant of cell envelope integrity and virulence. J. Bacteriol. 201:e00134-19. doi: 10.1128/JB.00134-19, PMID: 30936371 PMC6531618

[ref17] Hop H. T. Huy T. X. N. Lee H. J. Kim S. (2023). Intracellular growth of Brucella is mediated by Dps-dependent activation of ferritinophagy. EMBO Rep. 24:e55376. doi: 10.15252/embr.202255376, PMID: 37503678 PMC10481649

[ref18] Hovingh E. S. van den Broek B. Jongerius I. (2016). Hijacking complement regulatory proteins for bacterial immune evasion. Front. Microbiol. 7:2004. doi: 10.3389/fmicb.2016.02004, PMID: 28066340 PMC5167704

[ref19] Huy T. X. N. Nguyen T. T. Reyes A. W. B. Vu S. H. Min W. Lee H. J.. (2021). Immunization with a combination of four recombinant *Brucella abortus* proteins Omp 16, Omp 19, Omp 28, and L7/L12 induces T helper 1 immune response against virulent *B. abortus* 544 infection in BALB/c mice. Front. Vet. Sci. 7:577026. doi: 10.3389/fvets.2020.577026, PMID: 33553273 PMC7854899

[ref20] Im Y. B. Park W. B. Jung M. Kim S. Yoo H. S. (2018). Comparative analysis of immune responses to outer membrane antigens OMP10, OMP19, and OMP28 of *Brucella abortus*. Jpn. J. Infect. Dis. 71, 197–204. doi: 10.7883/yoken.JJID.2017.019, PMID: 29709972

[ref21] Jin X. Gou Y. Xin Y. Li J. Sun J. Li T.. (2023). Advancements in understanding the molecular and immune mechanisms of Bartonella pathogenicity. Front. Microbiol. 14:1196700. doi: 10.3389/fmicb.2023.1196700, PMID: 37362930 PMC10288214

[ref22] Juncker A. S. Willenbrock H. von Heijne G. Brunak S. Nielsen H. Krogh A. (2003). Prediction of lipoprotein signal peptides in Gram-negative bacteria. Protein Sci. 12, 1652–1662. doi: 10.1110/ps.0303703, PMID: 12876315 PMC2323952

[ref23] Kaushik P. Singh D. K. Kumar S. V. Tiwari A. K. Shukla G. Dayal S.. (2010). Protection of mice against *Brucella abortus* 544 challenge by vaccination with recombinant OMP28 adjuvanted with CpG oligonucleotides. Vet. Res. Commun. 34, 119–132. doi: 10.1007/s11259-009-9337-x, PMID: 20013309

[ref24] Kim S. Watanabe K. Shirahata T. Watarai M. (2004). Zinc uptake system (znuA locus) of *Brucella abortus* is essential for intracellular survival and virulence in mice. J. Vet. Med. Sci. 66, 1059–1063. doi: 10.1292/jvms.66.1059, PMID: 15472468

[ref25] Kitaura H. Kinomoto M. Yamada T. (1999). Ribosomal protein L7 included in tuberculin purified protein derivative (PPD) is a major heat-resistant protein inducing strong delayed-type hypersensitivity. Scand. J. Immunol. 50, 580–587. doi: 10.1046/j.1365-3083.1999.00630.x, PMID: 10607306

[ref26] Krogh A. Larsson B. von Heijne G. Sonnhammer E. L. (2001). Predicting transmembrane protein topology with a hidden Markov model: application to complete genomes. J. Mol. Biol. 305, 567–580. doi: 10.1006/jmbi.2000.4315, PMID: 11152613

[ref27] Mahajan N. K. Kulshreshtha R. C. Malik G. Dahiya J. P. (2005). Immunogenicity of major cell surface protein(s) of *Brucella melitensis* Rev 1. Vet. Res. Commun. 29, 189–199. doi: 10.1023/b:verc.0000047500.20855.7a, PMID: 15736854

[ref28] Malik M. Khan S. Ullah A. Hassan M. Haq M. U. Ahmad S.. (2023). Proteome-wide screening of potential vaccine targets against *Brucella melitensis*. Vaccines 11:263. doi: 10.3390/vaccines11020263, PMID: 36851141 PMC9966016

[ref29] Naganathan A. Wood M. P. Moore S. D. (2015). The large ribosomal subunit protein L9 enables the growth of EF-P deficient cells and enhances small subunit maturation. PLoS One 10:e0120060. doi: 10.1371/journal.pone.0120060, PMID: 25879934 PMC4399890

[ref30] Nandini P. Jakka P. Murugan S. Mazumdar V. Kumar D. Prakash R.. (2023). Immuno-profiling of Brucella proteins for developing improved vaccines and DIVA capable serodiagnostic assays for brucellosis. Front. Microbiol. 14:1253349. doi: 10.3389/fmicb.2023.1253349, PMID: 37860136 PMC10582347

[ref31] Naseer A. Mo S. Olsen S. C. McCluskey B. (2023). *Brucella melitensis* vaccines: a systematic review. Agriculture 13:2137. doi: 10.3390/agriculture13112137

[ref32] Oliveira S. C. Harms J. S. Banai M. Splitter G. A. (1996). Recombinant *Brucella abortus* proteins that induce proliferation and gamma-interferon secretion by CD4^+^ T cells from Brucella-vaccinated mice and delayed-type hypersensitivity in sensitized Guinea pigs. Cell. Immunol. 172, 262–268. doi: 10.1006/cimm.1996.0241, PMID: 8964089

[ref33] Pais T. F. Silva R. A. Smedegaard B. Appelberg R. Andersen P. (1998). Analysis of T cells recruited during delayed-type hypersensitivity to purified protein derivative (PPD) versus challenge with tuberculosis infection. Immunology 95, 69–75. doi: 10.1046/j.1365-2567.1998.00561.x, PMID: 9767459 PMC1364378

[ref34] Pandey S. Tripathi D. Khubaib M. Kumar A. Sheikh J. A. Sumanlatha G.. (2017). *Mycobacterium tuberculosis* peptidyl-prolyl isomerases are immunogenic, alter cytokine profile and aid in intracellular survival. Front. Cell. Infect. Microbiol. 7:38. doi: 10.3389/fcimb.2017.00038, PMID: 28261567 PMC5310130

[ref35] Petersen T. N. Brunak S. von Heijne G. Nielsen H. (2011). Signal P 4.0: discriminating signal peptides from transmembrane regions. Nat. Methods 8, 785–786. doi: 10.1038/nmeth.1701, PMID: 21959131

[ref36] Poetsch A. Marchesini M. I. (2020). Proteomics of Brucella. Proteomes 8:8. doi: 10.3390/proteomes8020008 32331335 PMC7355872

[ref37] Qureshi K. A. Parvez A. Fahmy N. A. Abdel Hady B. H. Kumar S. Ganguly A.. (2023). Brucellosis: epidemiology, pathogenesis, diagnosis and treatment-a comprehensive review. Ann. Med. 55:2295398. doi: 10.1080/07853890.2023.2295398, PMID: 38165919 PMC10769134

[ref38] Simborio H. L. T. Reyes A. W. B. Hop H. T. Arayan L. T. Min W. Lee H. J.. (2016). Immune modulation of recombinant Omp A against *Brucella abortus* 544 infection in mice. J. Microbiol. Biotechnol. 26, 603–609. doi: 10.4014/jmb.1509.09061, PMID: 26699748

[ref39] Sun D. Liu Y. Peng X. Dong H. Jiang H. Fan X.. (2023). Clp P protease modulates bacterial growth, stress response, and bacterial virulence in *Brucella abortus*. Vet. Res. 54:68. doi: 10.1186/s13567-023-01200-x, PMID: 37612737 PMC10464072

[ref40] Tibor A. Wansard V. Bielartz V. Delrue R. Danese I. Michel P.. (2002). Effect of omp 10 or omp 19 deletion on *Brucella abortus* outer membrane properties and virulence in mice. Infect. Immun. 70, 5540–5546. doi: 10.1128/IAI.70.10.5540-5546.2002, PMID: 12228280 PMC128365

[ref41] Tittarelli M. Atzeni M. Calistri P. Di Giannatale E. Ferri N. Marchi E.. (2015). Water buffaloes (*Bubalus bubalis*) vaccinated with *Brucella abortus* strain RB51 vaccine. Vet. Ital. 51, 99–105. doi: 10.12834/VetIt.472.2296.3, PMID: 26129660

[ref42] Verdiguel-Fernández L. Oropeza-Navarro R. Ortiz A. Robles-Pesina M. G. Ramírez-Lezama J. Castañeda-Ramírez A.. (2020). *Brucella melitensis* omp 31 mutant is attenuated and confers protection against virulent *Brucella melitensis* challenge in BALB/c mice. J. Microbiol. Biotechnol. 30, 497–504. doi: 10.4014/jmb.1908.08056, PMID: 31986561 PMC9728373

[ref43] Wang Z. Wang S. Wu Q. (2014). Cold shock protein a plays an important role in the stress adaptation and virulence of *Brucella melitensis*. FEMS Microbiol. Lett. 354, 27–36. doi: 10.1111/1574-6968.12430, PMID: 24661136

[ref44] Wareth G. Pletz M. W. Neubauer H. Murugaiyan J. (2020). Proteomics of Brucella: technologies and their applications for basic research and medical microbiology. Microorganisms 8:766. doi: 10.3390/microorganisms8050766, PMID: 32443785 PMC7285364

[ref45] WOAH (2022). “Brucellosis (infection with *B. abortus*, *B. melitensis* and *B. suis*) chapter 3.1.4 section. 3.1” in Manual of diagnostic tests and vaccines for terrestrial animals (Paris: WOAH).

[ref46] Yin R. Wang T. Dai H. Han J. Sun J. Liu N.. (2023). Immunogenic molecules associated with gut bacterial cell walls: chemical structures, immune-modulating functions, and mechanisms. Protein Cell 14, 776–785. doi: 10.1093/procel/pwad016, PMID: 37013853 PMC10599643

[ref47] Yu C. Chen Y. Lu C. Hwang J. (2006). Prediction of protein subcellular localization. Proteins 64, 643–651. doi: 10.1002/prot.21018, PMID: 16752418

[ref48] Yu C. Lin C. Hwang J. (2004). Predicting subcellular localization of proteins for gram-negative bacteria by support vector machines based on n-peptide compositions. Protein Sci. 13, 1402–1406. doi: 10.1110/ps.03479604, PMID: 15096640 PMC2286765

[ref49] Yu N. Y. Wagner J. R. Laird M. R. Melli G. Rey S. Lo R.. (2010). PSORTb 3.0: improved protein subcellular localization prediction with refined localization subcategories and predictive capabilities for all prokaryotes. Bioinformatics 26, 1608–1615. doi: 10.1093/bioinformatics/btq249, PMID: 20472543 PMC2887053

[ref50] Zhao Z. Yan F. Ji W. Luo D. Liu X. Xing L.. (2011). Identification of immunoreactive proteins of *Brucella melitensis* by immunoproteomics. Sci. China Life Sci. 54, 880–887. doi: 10.1007/s11427-011-4218-2, PMID: 21922434

[ref51] Zhu L. Wang Q. Wang Y. Xu Y. Peng D. Huang H.. (2020). Comparison of immune effects between Brucella recombinant Omp 10-Omp 28-L7/L12 proteins expressed in eukaryotic and prokaryotic systems. Front. Vet. Sci. 7:576. doi: 10.3389/fvets.2020.00576, PMID: 33195494 PMC7531237

